# Control of ADAM17 activity by regulation of its cellular localisation

**DOI:** 10.1038/srep35067

**Published:** 2016-10-12

**Authors:** Inken Lorenzen, Juliane Lokau, Yvonne Korpys, Mirja Oldefest, Charlotte M. Flynn, Ulrike Künzel, Christoph Garbers, Matthew Freeman, Joachim Grötzinger, Stefan Düsterhöft

**Affiliations:** 1 Institute of Biochemistry, Kiel University, Olshausenstr 40, 24098 Kiel, Germany; 2 Sir William Dunn School of Pathology, South Parks Road, Oxford OX1 3RE, UK

## Abstract

An important, irreversible step in many signalling pathways is the shedding of membrane-anchored proteins. A Disintegrin And Metalloproteinase (ADAM) 17 is one of the major sheddases involved in a variety of physiological and pathophysiological processes including regeneration, differentiation, and cancer progression. This central role in signalling implies that ADAM17 activity has to be tightly regulated, including at the level of localisation. Most mature ADAM17 is localised intracellularly, with only a small amount at the cell surface. We found that ADAM17 is constitutively internalised by clathrin-coated pits and that physiological stimulators such as GPCR ligands induce ADAM17-mediated shedding, but do not alter the cell-surface abundance of the protease. In contrast, the PKC-activating phorbol ester PMA, often used as a strong inducer of ADAM17, causes not only proteolysis by ADAM17 but also a rapid increase of the mature protease at the cell surface. This is followed by internalisation and subsequent degradation of the protease. Eventually, this leads to a substantial downregulation of mature ADAM17. Our results therefore imply that physiological activation of ADAM17 does not rely on its relocalisation, but that PMA-induced PKC activity drastically dysregulates the localisation of ADAM17.

Intercellular signalling is a fundamental process within multicellular organisms. A critical step in many signalling pathways is the proteolytic release of signalling molecules such as ligands or decoy receptors. Since proteolysis is an irreversible process this step has to be tightly regulated.

A Disintegrin And Metalloproteinase 17 (ADAM17) is a type-I transmembrane protease with a large ectodomain consisting of a prodomain, a catalytic domain, a disintegrin- and a membrane-proximal domain (MPD), as well as a short juxtamembrane segment called CANDIS followed by a transmembrane helix and a cytoplasmic region^1^,^2^,^3^. ADAM17 is critically involved in diverse signalling pathways controlling physiological and pathophysiological processes such as development, regeneration, immunity, (chronic) inflammation or tumourigenesis^4^,^5^,^6^,^7^,^8^. The involvement in such a variety of processes is based on a wide range of different ADAM17 substrates. More than 80 substrates have been identified so far, including many receptor ligands, the best studied of which are the pro-inflammatory cytokine tumour necrosis factor (TNF)-α^9^ and EGFR (epidermal growth factor receptor) ligands^8^,^10^. ADAM17-mediated shedding of cytokine receptors on the other hand causes desensitising of the cell to their ligands. Additionally, the liberated soluble receptor ectodomains can act either as inhibitors of signalling such as the interleukin-1 receptor II (IL-1R_II_)^11^ or as activators via trans-signalling like the interleukin-6 receptor (IL-6R)^12^,^13^.

Due to its central role in many signalling pathways, the activity of ADAM17 has to be tightly regulated. Excess ADAM17 activity leads to an increased release of EGFR ligands, which can drive tumour progression, whilst low ADAM17 activity can cause problems in development and regeneration due to decreased EGFR signalling^6^. Although many aspects of ADAM17 regulation have been studied, a complete picture is still missing.

Phorbol-12-myristat-13-acetat (PMA), a non-physiological PKC activator, is the strongest known and therefore often used stimulator of ADAM17-mediated shedding. However, several other stimuli are known which appear to be more physiological. For instance thrombin stimulates ADAM17-mediated shedding by activating the protease-activated receptor 1 (Par1), a G-protein coupled receptor (GPCR)^14^. Notably, it is still not clear which intracellular pathways are the physiological activators of ADAM17. Phosphorylation is a common way to regulate protein activity and Xu and Derynck^15^ showed that p38 MAPK is able to phosphorylate ADAM17 at T735 within the cytoplasmic region, resulting in ADAM17 activation.

In contrast, the finding that the cytoplasmic region is not required for the induction of shedding shifts the regulatory focus to the ectodomain^10^,^11^,^16^. Further studies show that the disulphide pattern of the MPD determines the ability of ADAM17 to bind and shed substrates, and that protein-disulphide isomerase (PDI) catalyses a change in the disulphide pattern resulting in an inactive ADAM17^2^,^3^,^17^,^18^.

Recent studies established a new model of ADAM17 activation. Activators of ADAM17 induce phosphatidylserine exposure at the outer leaflet of the cell membrane, which causes ADAM17 to bind to the membrane via its MPD and CANDIS and thereby facilitates the shedding process^19^,^20^.

Another level of regulation is the cellular localisation of ADAM17. A large amount of ADAM17 is stored within the endoplasmic reticulum (ER) as the immature proform/zymogen with the prodomain still covalently attached. The maturation of ADAM17 takes place in the Golgi apparatus, where the prodomain is cleaved off by pro-protein convertases such as Furin^21^. Essential components of the forward trafficking of ADAM17 from the ER to the Golgi apparatus are iRhom1 and iRhom2^22^,^23^,^24^. A knockout of both iRhoms causes the loss of ER to Golgi trafficking of ADAM17 and consequent lack of its maturation and loss of ADAM17-mediated shedding^23^. Furthermore, it is still not clear if shedding only takes place on the cell surface or if it also can occur in intracellular vesicles.

Previous studies have shown different effects of PMA treatment on ADAM17 localisation. Soond, *et al*.^25^ reported an increased surface expression of overexpressed ADAM17 and a subsequent decrease to basal levels within minutes. In contrast, others showed a downregulation of mature ADAM17 in the cell and on the surface^21^,^26^. The use of hypertonic medium prevented the PMA-induced downregulation of ADAM17 on the cell surface^26^. However, there are different internalisation pathways, which can be divided into the clathrin-dependent and clathrin-independent pathways, e.g. the caveolin-dependent endocytosis^27^. Since hypertonic conditions inhibit internalisation in general^28^, it is still unknown which pathway is used to internalise ADAM17.

Furthermore, it was recently discovered that ADAM17 undergoes PACS-2-dependent endocytic recycling under unstimulated conditions, and that the knockout of PACS-2 in mice leads to impaired recycling, favouring the degradation of ADAM17^29^.

We have investigated the details of ADAM17 internalisation and whether this process is caused by ADAM17 activation and hence presents a mode of inactivation. We found that ADAM17 undergoes constitutive clathrin-dependent internalisation and that this turnover is not linked to ADAM17 activation by physiological stimuli. We compared the activation, cell surface expression, and internalisation of ADAM17 in response to different stimuli such as PMA and GPCR ligands. The physiological stimulators thrombin and histamine had no detectable effect on the localisation of ADAM17. In contrast to the GPCR ligands, PMA induced a rapid increase of endogenous, mature ADAM17 on the cell surface of HEK293 and HeLa cells, followed by internalisation and lysosomal degradation of the protease. Inhibition of the clathrin-dependent endocytosis slowed down the internalisation process, but was unable to prevent it. Eventually, stimulation with PMA emptied the whole pool of mature ADAM17 within hours without affecting the pool of immature ADAM17. A full recovery of the mature ADAM17 pool was not observed even after 24 hours. We further overexpressed iRhom1 in order to accelerate ER to Golgi trafficking and subsequently ADAM17 maturation, but this did not prevent the disappearance of the mature protease.

In summary, we report that different stimuli cause different modes of activation, internalisation and degradation of ADAM17, which has implications not only for our understanding of physiological and pathophysiological signalling, but also for common methods for studying this important signal regulator.

## Results

### Stimulation of ADAM17-mediated shedding is not directly linked to degradation of the protease

To address the question whether activation of ADAM17-mediated shedding causes a subsequent decrease of the mature form of the protease, we tested different activators.

**Figure 1:**
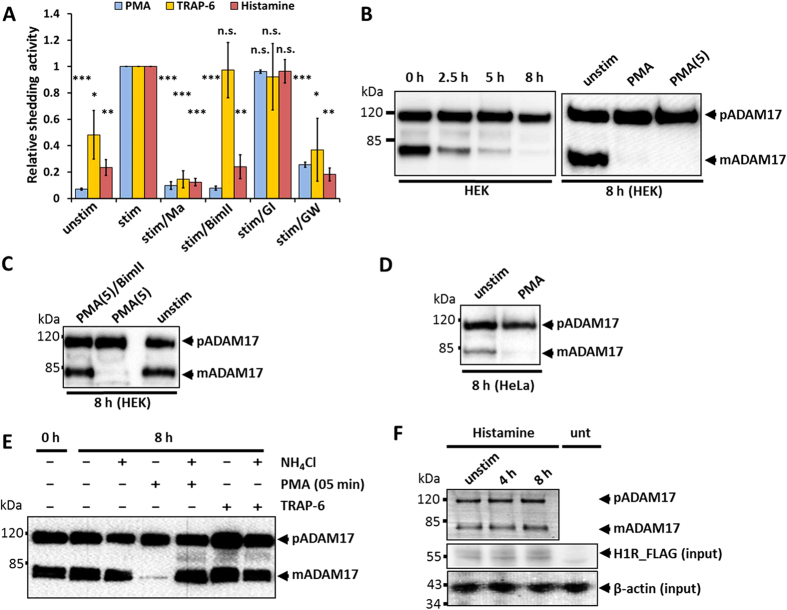
Effect of different stimulators on the activation and degradation of mature ADAM17. (**A**) HEK293 cells were transfected with AP_IL-1R_II_ or transfected with AP_IL-1R_II_ and H1R. Shedding activity was measured after a 30-minute treatment with solvent/DMSO (unstim), with a stimulator (stim) or with a stimulator and an inhibitor. 100 nM PMA, 10 μM Histamine or 30 μM thrombin receptor agonistic peptide (TRAP-6) SFLLRN were used as stimulators. For inhibition cells were treated with following compounds: 10 μM metalloprotease inhibitor marimastat (Ma), 5 μM PKC inhibitor BimII, 1 μM ADAM10 inhibitor GI or 1 μM ADAM10/17 inhibitor GW. All values were normalised to the stimulated values. n = 3; *p < 0.05, **p < 0.01, ***p < 0.001. (**B**–**F**) Glycosylated proteins were enriched by precipitation with ConA-Sepharose and immunoblotted (n = 3). (**B**) Left panel: Influence of PMA on mature ADAM17 (mADAM17) and its proform (pADAM17) were analysed in HEK293 2.5, 5 or 8 hours after PMA stimulation. Right panel: Cells were incubated with 100 nM PMA either for the whole 8 hours (PMA) or only for 5 minutes with a washing step and incubation in media without stimulator afterwards (PMA (5)). (**C**) Cells were incubated with 100 nM PMA for 5 minutes and incubated for 8 hours without PMA or equally stimulated and simultaneously treated with BimII. (**D**) Influence of 100 nM PMA on HeLa cells. (**E**) Influence of PAR1 activation (30 μM TRAP-6) on ADAM17 degradation. To inhibit lysosomal degradation 50 mM NH_4_Cl were used. (**F**) HEK293 cells were transfected with H1R and were not stimulated or stimulated with histamine for 4 or 8 hours. unt: untransfected control lysate.

As expected, PMA induced ADAM17-mediated shedding of the AP-tagged IL-1R_II_, which was transiently transfected into HEK293 cells (Fig. 1A). IL-1R_II_ shedding was inhibited by the metalloprotease inhibitor marimastat (Ma), the ADAM10/ADAM17 inhibitor GW and the pan-PKC inhibitor BimII but not by the ADAM10 inhibitor GI. Consistent with a previous report^30^, PMA decreased the amount of mature ADAM17 (mADAM17) but not of its proform (pADAM17) in HEK293 cells (Fig. 1B) within hours. However, no downregulation was detected within the first 30 minutes after PMA treatment (Suppl. Fig. 1A). Eight hours after stimulation with PMA most of the mature ADAM17 was gone and this downregulation was induced either by incubating the cells with PMA for the whole time (long-term treatment) or by a short-term stimulation of cells for only five minutes with a subsequent washing step followed by incubation in PMA-free medium (Fig. 1B). The process was blocked by pre-treatment of the cells with BimII (Fig. 1C), implying a role for PKC in loss of mature ADAM17. This effect was not restricted to HEK293 cells, as PMA also induce the downregulation of mature ADAM17 in HeLa cells (Fig. 1D).

Next, we tested a physiological stimulator for ADAM17-mediated shedding on HEK293 cells^14^, the thrombin receptor agonistic peptide (TRAP-6) SFLLRN, which specifically activates PAR1. As shown in Fig. 1A, IL-1R_II_ shedding was induced by TRAP-6 treatment of HEK293 cells expressing AP-tagged IL-1R_II_; this was blocked by Ma and GW, but not by GI or BimII. Although ADAM17-mediated shedding was stimulated by TRAP-6 there was no change in the amount of mature ADAM17 in HEK293 cells after eight hours (Fig. 1E). Similarly, treatment with NH_4_Cl, which prevents lysosomal degradation, did not affect the amount of mature ADAM17 in cells either left unstimulated or stimulated with TRAP-6, but completely blocked PMA-induced degradation (Fig. 1E).

Since TRAP-6-stimulated ADAM17 activation is PKC independent, we used histamine to test if PKC-dependent signalling activated by stimulation of the GPCR histamine H1 receptor (H1R) is sufficient for ADAM17 downregulation. To induce ADAM17-mediated shedding of AP-IL-1R_II_ with histamine, HEK293 cells were co-transfected with H1R. The histamine-stimulated shedding was blocked by Ma and GW but not by GI (Fig. 1A), proving the involvement of ADAM17 but not ADAM10. Additionally, BimII inhibited histamine-induced ADAM17-mediated shedding (Fig. 1A). As with thrombin-mediated activation, although histamine-induced ADAM17-activation was PKC-dependent, it did not induce a reduction of mature ADAM17 (Fig. 1F). We also tested whether higher concentrations of both physiological stimulators had an effect on ADAM17. However, higher concentrations than used before had an effect neither on the shedding activity nor on the amount of mature ADAM17 (Suppl. Fig. 1B–E). Taken together, these results show that, while all three stimulators induce ADAM17 activity, they have different influences on the degradation of the protease, proving that pharmacological stimulation does not induce the normal physiological mechanism, and that lysosomal degradation is not a necessary consequence of induced ADAM17 activity.

### PMA-induced downregulation of mature ADAM17 abolishes shedding activity

**Figure 2:**
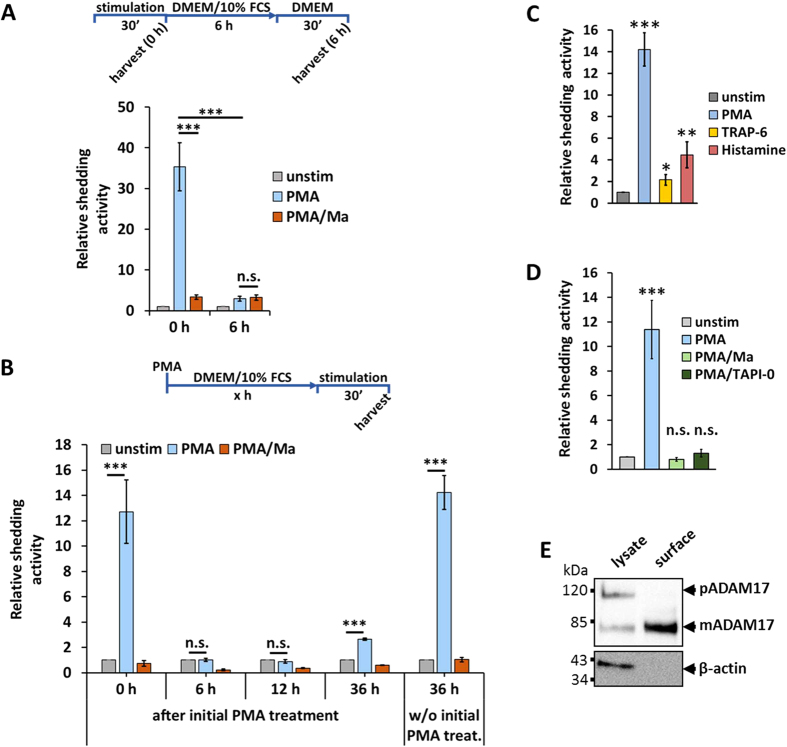
Effect of PMA on ADAM17-mediated shedding. (**A**) HEK293 cells were transfected with AP_IL-1R_II_. Shedding activity was measured after a 30-minute treatment with solvent/DMSO (unstim), with 100 nM PMA or with 100 nM PMA and 10 μM marimastat (Ma). Additionally, 6 hours after the first measurement the supernatant was changed and again collected after 30 minutes without additional stimulation and shedding activity was measured again. 100 nM PMA was used as stimulator and for inhibition cells were treated with 10 μM metalloprotease inhibitor marimastat (Ma). All values were normalised to the unstimulated values. n = 4; *p < 0.05, ** p < 0.01, *** p < 0.001. (**B**) HEK293 cells were transfected with AP_IL-1R_II_. Cells were treated with 100 nM PMA and 10 μM marimastat to block the loss of substrate. After indicated time, shedding activity was measured after a 30-minute treatment with solvent/DMSO (unstim), with 100 nM PMA or with 100 nM PMA and 10 μM marimastat (Ma). All values were normalised to the unstimulated values. n = 4; *p < 0.05, **p < 0.01, ***p < 0.001. (**C**) ADAM17 activity comparison of the different stimulators normalised to the unstimulated (unstim) samples. n = 3; *p < 0.05, **p < 0.01, ***p < 0.001. (**D**) HEK293 cells were transfected with AP_IL-1R_II_. Shedding activity was measured after a 30-minute treatment with solvent/DMSO (unstim), with PMA or with PMA and an inhibitor. 100 nM PMA was used as stimulator. For inhibition, cells were treated with following compounds: 10 μM membrane-permeable metalloprotease inhibitor marimastat (Ma) or 15 μM non-membrane permeable metalloprotease inhibitor TAPI-0. All values were normalised to the unstimulated values. n = 4; *p < 0.05, **p < 0.01, ***p < 0.001. (**E**) Western blot of HEK293 lysate and HEK293 cell-surface proteins (surface biotinylation). n = 3.

To investigate the long-term effect of PMA on the ADAM17 activity we first examined whether shedding is still detectable six hours after PMA stimulation (Fig. 2A). After this timeframe, no ADAM17-mediated shedding was observed. An additional experiment revealed that after an initial treatment with PMA and marimastat (to block the loss of substrate in the cell) ADAM17-mediated shedding was no longer inducible 6 hours or 12 hours after PMA treatment (Fig. 2B). A recovery of ADAM17-mediated shedding was detected 36 hours after the initial PMA stimulation. These data show that the PMA-induced downregulation decreases the amount of mature ADAM17 not only below the detection level of Western blotting, but also below the level of detectable shedding activity.

### PMA-induced PKC activation influences ADAM17 surface expression, whilst stimulation of PAR1 does not

Interestingly, the ADAM17 activity stimulated by PMA is three to four times higher than the activity induced with histamine or TRAP-6 (Fig. 2C). To determine whether the PMA-induced shedding solely occurs at the cell surface, we used the ADAM17 inhibitor TAPI-0. In contrast to marimastat, TAPI-0 is not membrane permeable and hence only inhibits shedding at the cell surface. Both inhibitors block shedding equally (Fig. 2D) which shows that shedding only takes place at the cell surface. Since the shedding process takes place at the cell surface, we investigated whether different stimulators lead to different ADAM17 surface expression and thereby subsequently distinct shedding activities (Fig. 2C).

We first verified that the surface-protein fraction obtained by surface biotinylation of HEK293 cells solely contained mature ADAM17, which is in line with previous studies^26^,^31^ (Fig. 2E).

**Figure 3:**
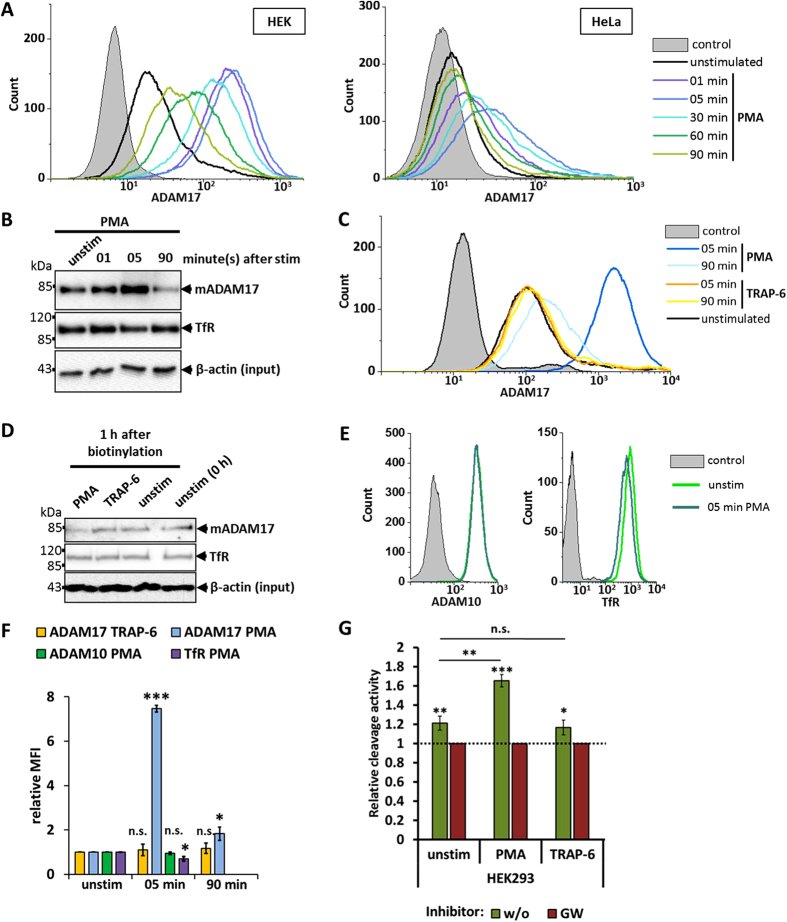
Surface expression of ADAM17 after stimulation. (**A**) HEK293 and HeLa cells were either left unstimulated or were stimulated with 100 nM PMA. At given time points cells were harvested and stained with anti-ADAM17 (A300E) for flow-cytometry analysis. Each depicted flow cytometry graph is one representative experiment of a set of n = 3. (**B**) Western blot of cell-surface proteins of HEK293 cells, which were left unstimulated or stimulated with 100 nM PMA. Cell-surface biotinylation was performed at given times after stimulation. The lysates, which were used for the Streptavidin-Sepharose precipitation were immunoblotted as additional input control. n = 3. (**C**) HEK293 cells were treated with 100 nM PMA, 30 μM TRAP-6 or left unstimulated. At given time points cells were harvested and stained with anti-ADAM17 (A300E) for flow-cytometry analysis. Each depicted flow cytometry graph is one representative experiment of a set of n = 3. (**D**) Pulse-chase experiments were performed with HEK293 cells. Surface proteins were biotinylated and immediately harvested (0 h) or biotinylated and incubated for 1 hour with solvent/DMSO (unstim), with 30 μM TRAP-6 or with 100 nM PMA. Biotinylated proteins were immunoblotted (n = 3). The lysates, which were used for the Streptavidin-Sepharose precipitation were immunoblotted as additional input control. (**E**) Flow cytometry of cells stimulated with PMA for 5 minutes or left unstimulated (unstim). Cells were stained for either ADAM10 or Transferrin receptor (TfR). Depicted flow cytometry graph is one representative experiment of a set of n = 3. (**F**) Comparison of relative MFI (median fluorescence intensity) of flow cytometry analysis (**C**) and (**E**). The MFIs were normalised to the respective unstimulated sample. *p < 0.05, **p < 0.01, ***p < 0.001. (**G**) Peptide-substrate cleavage was performed with HEK293 cells. Cells were treated with or without GW and incubated with solvent/DMSO (unstim), PMA or TRAP-6. For each stimulator or DMSO the cleavage activity was normalised to the GW treated sample. n = 3 *p < 0.05, **p < 0.01, ***p < 0.001.

To get a detailed insight into the fate of ADAM17, its expression at the cell surface of HEK293 and HeLa cells was analysed by flow cytometry at different time points after stimulation with PMA. In HEK293 cells, a drastic increase of mature ADAM17 within less than five minutes was observed (Fig. 3A) without a change in the total amount of ADAM17 in the cell (Suppl. Fig. 2A), followed by a constant slow decrease over 90 minutes. The same overall trend of rapid increase and slow decrease was observed by using biotinylation of surface proteins at different time points (Fig. 3B). Additionally, flow cytometry analysis of HeLa cells showed a similar picture of fast increase and subsequent decrease of ADAM17 surface expression upon stimulation with PMA (Fig. 3A).

Having shown that ADAM17 is activated via PAR1, we sought to investigate how TRAP-6 stimulated PAR1-activation influences expression of ADAM17 at the cell surface. In contrast to PMA treatment, the stimulation of HEK293 cells with different concentrations of TRAP-6 did not result in any difference in the amount of ADAM17 on the surface after five or 90 minutes (Fig. 3C,F; Suppl. Fig. 2B). One explanation for the constant level of cell-surface ADAM17 after TRAP-6 stimulation would be that new ADAM17 molecules are transported to the cell surface to balance TRAP-6 induced internalisation and degradation. To check whether such a turnover is promoted by TRAP-6 a pulse chase experiment was performed. Cell-surface proteins of HEK293 cells were biotinylated and subsequently the cells were treated with TRAP-6 or PMA. One hour after stimulation the amount of mature ADAM17 was unchanged in TRAP-6 treated cells, whilst in PMA-treated cells the amount was decreased (Fig. 3D). This indicates that, unlike PMA stimulation, activation of ADAM17 via PAR1 does not induce a change in the cell-surface expression of the protease.

In order to unravel whether increased cell-surface expression after PMA stimulation is specific for ADAM17, we stimulated HEK293 cells with PMA for five minutes and stained for ADAM10 and the transferrin receptor (TfR). Unlike ADAM17, ADAM10 and TfR did not show a higher surface expression five minutes after stimulation with PMA (Fig. 3E,F). Instead, the amount of surface TfR was slightly decreased (Fig. 3E,F).

For the shedding of membrane-bound substrates, ADAM17 has to change its conformation. Activators of shedding lead to an exposure of phosphatidylserine on the outer layer of the cell membrane providing an interaction hub for the ADAM17 ectodomain^20^. Binding of parts of the ectodomain brings the catalytic site of ADAM17 in close proximity to the cleavage site of membrane-bound substrate and thereby enables the shedding process. In contrast, cleavage of soluble peptide substrates does not require this process. Therefore, peptide substrates represent a useful tool to monitor the amount of active ADAM17 on the cell surface independent of the actual shedding process. We used a fluorogenic peptide which is derived from the ADAM17 cleavage site of TNF-α and therefore not cleavable by ADAM10^32^ to test the effect of PMA and TRAP-6 on the amount of active ADAM17 on HEK293 cells. GW was used to block the cleavage of the peptide. In line with the previous results TRAP-6 does not increase the amount of active ADAM17 on the surface, while PMA does (Fig. 3G).

Collectively, these results suggest that increased ADAM17 expression and activity at the cell surface after PMA treatment is caused by the activation of specific PKC signalling pathways, distinct from those triggered by PAR1; they also imply that internalisation and degradation are not a necessary consequence of ADAM17 proteolytic activation.

### ADAM17 is mainly endocytosed via a clathrin-dependent pathway

**Figure 4:**
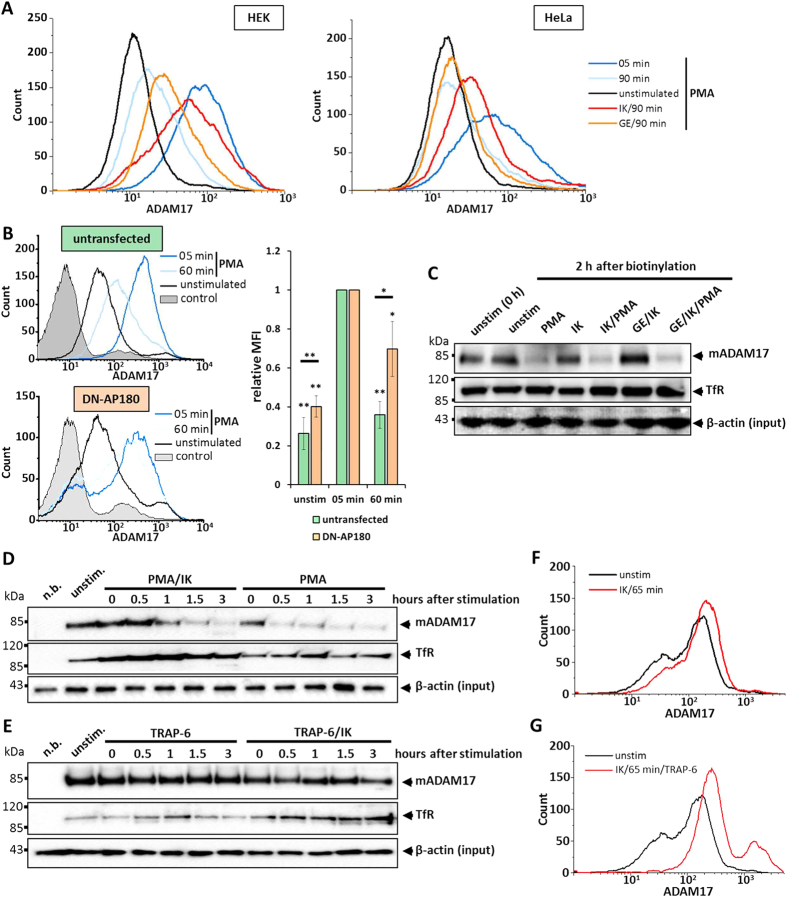
Inhibition of ADAM17 internalisation. (**A**–**G**) As inhibitor against the clathrin-dependent internalisation 5 μM ikarugamycin (IK) were used. To inhibit caveolin-dependent internalisation cells were treated with 200 μM genistein (GE). Each depicted flow cytometry graph is one representative experiment of a set of n = 3. (**A**) Flow-cytometry analysis of HEK293 and HeLa cells, which were left unstimulated, were stimulated with 100 nM PMA or were stimulated with 100 nM PMA and treated either with genistein or with ikarugamycin 1 hour before stimulation. (**B**) HEK293 cells were left untransfected or were transfected with DN-AP180_BFP2. Afterwards, cells were stimulated with PMA for 5 minutes or 60 minutes, or left unstimulated and harvested for flow cytometry analysis. Each depicted flow cytometry graph is one representative experiment of a set of n = 4. Right panel: Comparison of relative MFI (median fluorescence intensity) between untransfected and with DN-AP180 transfected cells. The MFIs were normalised to the respective 5 minutes stimulated sample. (**C**) Pulse chase experiments: HEK293 cells were incubated with inhibitors like described before. After 1 hour surface proteins were biotinylated and cells were immediately harvested (unstim (0 h)), or were stimulated with 100 nM PMA or left unstimulated in addition to the indicated treatment with inhibitors for 2 hours. The lysates, which were used for the Streptavidin-Sepharose precipitation were immunoblotted as additional input control. (**D**,**E**) Western blots of cell-surface fractions of HEK293 cells, which were either left unstimulated, stimulated with 100 nM PMA (**D**) or stimulated with 30 μM TRAP-6 (**E**). An additional set of cells were treated with ikarugamycin and were stimulated like described before 1 hour after treatment with the inhibitor. Cell-surface proteins were biotinylated at given time points after stimulation. The lysates, which were used for the Streptavidin-Sepharose precipitation were immunoblotted as additional input control. (**F**) Flow-cytometry analysis of HEK293 cells, which were left unstimulated and were treated with ikarugamycin for indicated times. (**G**) Flow-cytometry analysis of HEK293 cells, which were treated with ikarugamycin for 60-minute prior stimulation. Cells were analysed 5 minutes after stimulation with 30 μM TRAP-6.

Which endocytic pathway is responsible for ADAM17 internalisation? Its cytoplasmic region contains a potential motif for clathrin-dependent internalisation (Y702-E703-S704-705L), so we investigated whether ADAM17 is endocytosed via a clathrin-dependent or independent pathway. Flow cytometry analysis of HEK293 and HeLa cells stimulated with PMA and treated with ikarugamycin, an inhibitor of clathrin-dependent endocytosis, revealed that ikarugamycin inhibited PMA-induced internalisation (Fig. 4A). We confirmed this finding by overexpression of the dominant negative (DN) form of the clathrin adapter protein AP180, which inhibits the formation of clathrin-coated pits^33^. PMA induced a strong increase in ADAM17 cell-surface expression irrespective of DN-AP180 expression, but in contrast to untransfected cells, ADAM17 stayed at the cell surface when DN-AP180 was overexpressed (Fig. 4B).

Interestingly, an additional pulse chase experiment in stimulated HEK293 cells showed that there was no rescue of mature ADAM17 by treating cells with ikarugamycin compared to cells incubated without the inhibitor (Fig. 4C). Furthermore, surface biotinylation at different time points after stimulation with PMA showed that eventually the amount of surface ADAM17 is drastically decreased with or without inhibiting clathrin-dependent endocytosis, although mature ADAM17 was detected on the cell surface for a longer time with inhibitor (Fig. 4D). Taken together, these data indicate that inhibition of clathrin-dependent internalisation decelerates, but does not prevent, endocytosis of ADAM17. Notably, the transferrin receptor, which is an archetypical cargo for clathrin-dependent internalisation and recycling^34^, was enriched on the surface when cells were treated with ikarugamycin (Fig. 4D,E).

In contrast, cells stimulated with TRAP-6 did not show a decrease in the amount of surface ADAM17 irrespective of whether clathrin-dependent endocytosis was inhibited (Fig. 4E). Again, cells treated with ikarugamycin showed a higher surface expression of the transferrin receptor (Fig. 4E).

Since ADAM17 undergoes recycling in unstimulated cells^29^, we examined whether this recycling is clathrin-dependent. We treated unstimulated cells with ikarugamycin and analysed changes of cell-surface ADAM17 by flow cytometry. As shown in Fig. 4F, the inhibition of clathrin-dependent endocytosis led to an increase of ADAM17 on the cell surface. We performed the same experiment, but treated the cells with TRAP-6 in addition to ikarugamycin. However, as seen above, activation of PAR1 had no influence on the cell-surface localisation of ADAM17 compared to unstimulated cells (Fig. 4G). Furthermore, genetic inhibition of clathrin-coated pits formation by DN-AP180 also shows an increase in the abundancy of cell-surface ADAM17 on unstimulated cells (Fig. 4B).

ADAM17 has been described to be associated with lipid rafts^35^ and especially with caveolin-rich membrane domains^36^,^37^. Since PMA-induced internalisation of ADAM17 is not solely clathrin dependent, we investigated whether inhibition of caveolin-dependent internalisation by genistein influences the cell-surface expression of ADAM17. HEK293 cells treated with genistein showed a higher surface expression of ADAM17 90 minutes after stimulation with PMA compared to cells incubated without inhibitor. This effect was not observed in HeLa cells (Fig. 4A).

Since ikarugamycin and genistein can each reduce PMA-induced internalisation of ADAM17, we tested whether a combination of both inhibitors is sufficient to completely block ADAM17 internalisation. A pulse chase experiment with HEK293 cells showed no rescue of biotinylated surface ADAM17 when both inhibitors were used compared to the treatment with only ikarugamycin (Fig. 4C).

The cytoplasmic region of ADAM17 contains the putative binding motif YXXØ (Y702-E703-S704-L705) for AP-2, an important adapter protein for clathrin-dependent internalisation^38^. To test whether this motif is crucial for the internalisation of ADAM17, we used a HEK293 cell line stably expressing an ADAM17 variant with two point mutations within this motif called ADAM17_YALT (Y702A, L705T). A disrupted forming of clathrin-coated pits attached to ADAM17_YALT should lead to an impaired internalisation and subsequent degradation of this variant. However, a pulse-chase experiment revealed no difference between wild-type ADAM17 and ADAM17_YALT (Suppl. Fig. 3).

Altogether, our data suggest that ADAM17 is constitutively internalised via the clathrin-dependent pathway in unstimulated cells and that physiological activation of the protease does not alter this process. However, PMA-induced internalisation of ADAM17 is mediated in part via clathrin-dependent as well as caveolin-dependent pathways. Interestingly, inhibition of both does not completely abrogate PMA-induced internalisation, which points towards further, so far unknown, mechanisms.

### Overexpression of iRhom1 does not prevent PMA-induced downregulation of mature ADAM17 nor accelerate its recovery

**Figure 5:**
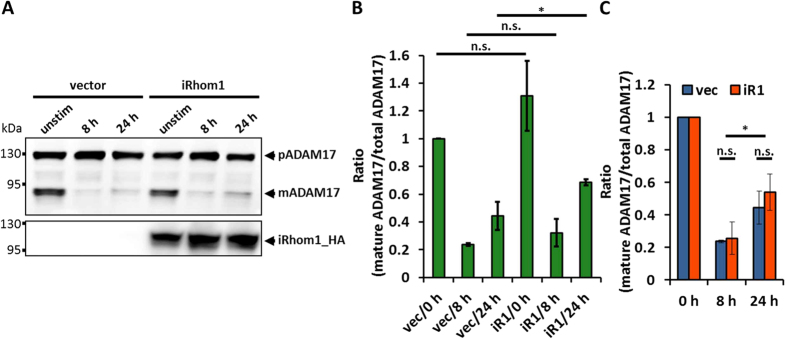
Overexpression of iRhom1 does not prevent the depletion of mature ADAM17. (**A**) The influence of PMA on ADAM17 in HEK293 cells overexpressing iRhom1 was analysed. Therefore, cells were transfected either with an empty vector (vec) or with iRhom1_M6P (iR1). After 48 hours cells were left unstimulated (unstim) or were stimulated with 100 nM PMA for 5 minutes. 8 hours or 24 hours after PMA stimulation the cells were harvested. Glycosylated proteins were enriched by precipitation with ConA-Sepharose and immunoblotted. (**B**,**C**) Amount of mature ADAM17 and its proform was measured by densitometry of immunoblots (A). The results are mean values of three independent experiments. (**B**) Shown are the ratios of mature ADAM17 to the total amount of ADAM17 normalised to unstimulated and untransfected cells (vec/0 h). (**C**) Comparison of the rate of downregulation and recovery. Each setup (vector and iRhom1 overexpression) was normalised to its initial amount of mature ADAM17 (0 h).

Although the pool of mature ADAM17 is drastically downregulated after stimulation with PMA, the pool of immature ADAM17 seems not to change, suggesting that it should be able to provide a source of new mature ADAM17. To examine the recovery of mature ADAM17, we looked at the ratio of mature ADAM17 to the total amount of ADAM17 in the cell at zero hours, eight hours and 24 hours after stimulation with PMA (Fig. 5A). 24 hours after stimulation some recovery of mature ADAM17 was observed in HEK293 cells. Nevertheless, the amount of mature ADAM17 was still only 45 ± 10% of the initial amount (Fig. 5B).

Since iRhoms are important factors for the forward trafficking of ADAM17 from the ER to the Golgi apparatus^22^,^23^, where maturation takes place, we tested whether overexpression of iRhom1 can accelerate the recovery of the pool of mature ADAM17. Indeed, we observed a significantly higher amount of mature ADAM17 (69 ± 2%) in iRhom1 overexpressing cells after 24 hours compared to cells without overexpression (Fig. 5B). However, when the actual rates of the downregulation or the recovery were compared between untransfected and iRhom1 overexpression cells, there was no significant difference (Fig. 5C). Taken together, these results suggest that an overexpression of iRhom1 does not influence the PMA-induced degradation or the subsequent reappearance of mature ADAM17.

## Discussion

ADAM17 is a crucial part of many signalling pathways and thereby orchestrates many physiological but also pathophysiological processes. In order to keep the balance between both sides ADAM17 activity has to be tightly regulated. One important mode of regulation is the actual activation of the shedding process, which was recently shown to involve an exposure of phosphatidylserine at the outer layer of the cell membrane. This leads to a conformational change in the ectodomain of ADAM17 moving its catalytic site to the cleavage sites of the transmembrane-anchored substrates^19^,^20^. The intensity of an enzymatic activity is, among others, dependent on the availability of the enzyme. Since ADAM17-mediated shedding takes place at the cell surface, one important level of regulation is the localisation of the mature and therefore activatable ADAM17. There are two ways to control the surface abundancy of a protein. One is the regulation of the transport to the surface and the other is the removal from the cell surface, e.g. by internalisation.

In this study, we show for the first time that endogenous ADAM17 is constitutively internalised by clathrin-coated pits and that activation of ADAM17 by physiological stimuli does not alter the abundancy of the mature protease on the surface or alter the speed of its turnover.

A mutation of the putative AP-2 binding motif (Y702-E703-S704-L705) within the cytoplasmic region of ADAM17 does not impair constitutive internalisation of the protease. This finding indicates that this motif is not required for an efficient constitutive internalisation. However, since this experiment was performed in a stably transfected cell line overexpressing the ADAM17 variant, we cannot rule out an effect of the overexpression on trafficking of the protease.

**Figure 6:**
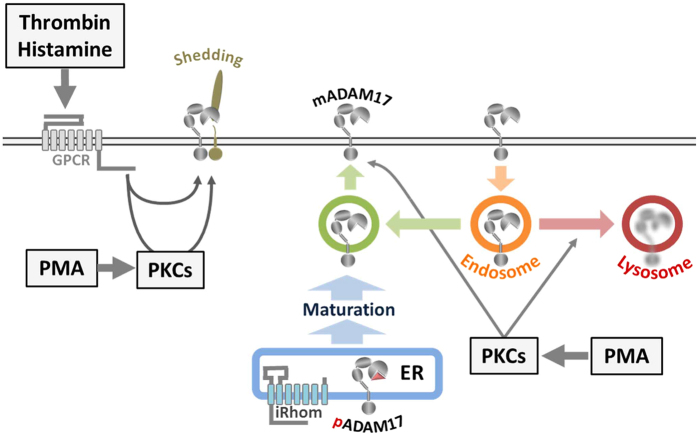
Model of the ADAM17 life cycle. ADAM17 exists as immature proform (pADAM17) and as mature protease (mADAM17) in cells. After ADAM17 is translated into the ER, it travels iRhom-dependent to the Golgi apparatus, where the maturation takes place. This process seems to be a tightly regulate bottleneck in the life cycle of ADAM17 dividing a big pool of immature from a smaller pool of mature molecules. Most of the mature ADAM17 seems to be intracellularly located whilst only a small amount is actually at the cell surface, where shedding can take place. Stimulation with physiological stimulators of ADAM17-mediated shedding such as Thrombin or Histamine does not alter the amount of cell surface ADAM17 or the overall amount of mature ADAM17 in the cell. Most recently it was shown, that most of the mature ADAM17 at the cell surface is internalised and recycled. The non-physiological PKC-activator PMA does not only activate ADAM17-mediated shedding but does also drastically dysregulate the localisation of mature ADAM17. PMA leads to a fast increase in surface expression followed by internalisation and lysosomal degradation of ADAM17. The activation of this path eventually empties the pool of mature ADAM17 within a few hours without affecting the pool of immature ADAM17.

In previous studies, internalisation of ADAM17 was described as an eventual consequence of ADAM17 activation^21^,^26^. However, in these studies the pharmacological activator PMA was used. This seeming contradiction to our findings implies other modes of action of PMA besides stimulation of ADAM17-mediated shedding (Fig. 6).

Our initial findings were consistent with the previous study that mature ADAM17 vanished after long-term treatment of cells with PMA^21^. We found that also a short-term treatment with PMA had the same effect and that this effect is PKC-dependent. Thereby, we ruled out that this process is driven by other PMA binding molecules such as Munc proteins, which are associated with vesicle formation^21^. Furthermore, our finding that treatment with a lysosomal inhibitor prevents the loss of mature ADAM17 demonstrates that PMA-activated signalling pathways direct the mature form of the protease to the lysosome where it is degraded.

Activation of GPCRs-mediated signalling, either PKC-dependent (histamine) or PKC-independent (TRAP-6), stimulated ADAM17-mediated shedding, but did not induce degradation of the protease. Although, in case of histamine, we cannot rule out that the overexpression of the histamine receptor H1R might have an effect on the cellular response. Whilst the stimulation with TRAP-6 did not alter the surface expression of ADAM17 on HEK293 cells, PMA induced a rapid increase in surface expression and a subsequent decrease below the basal level. Importantly, such a fast transport of proteins to the cell surface, within a few minutes, seems not to be a general response upon stimulation with PMA, since this effect was neither observable for ADAM10, the closest relative of ADAM17, nor for the transferrin receptor. Since PMA leads to a drastic increase in ADAM17 surface expression and activates the shedding process (Fig. 6), it explains why PMA is such a potent stimulator for ADAM17 compared to physiological stimuli.

We further demonstrated that the PMA-induced internalisation of cell-surface ADAM17 also occurred via clathrin-coated pits. However, the attempt to inhibit the clathrin-dependent endocytosis biochemically with ikarugamycin or genetically with DN-AP180 decelerated the internalisation of ADAM17, but did not prevent it. In contrast, we showed that the transferrin receptor, which is known to undergo clathrin-dependent recycling, was enriched on the cell surface. Furthermore, it was previously demonstrated that PMA induces the clathrin-dependent internalisation of the immunoglobulin CD4 which can be fully blocked by ikarugamycin^39^. These results indicate that there are additional processes that mediate PMA-induced decrease of surface ADAM17. We found that ADAM17 internalisation is marginally decreased when caveolin-dependent endocytosis is inhibited in HEK293 cells, which points towards a minor role of this pathway. We cannot exclude other endocytosis-independent mechanisms. One possibility is that ADAM17 itself is shed. However, previous experiments failed to find soluble ADAM17 in the supernatant^26^. Moreover, the fact that we can inhibit the downregulation with ammonium chloride, which is not known to inhibit shedding at the cell surface, also argues against this explanation. Another possibility is the formation of ADAM17 loaded exosomes. Recently, it was reported that ADAM17 can be found in exosomes^40^,^41^, and it would be still consistent with our findings, since ammonium chloride was also described to inhibit exosome formation^42^.

Overall, our results provide an explanation and clarification of previous studies, which appeared to contradict one another. Whilst Doedens and Black^26^ and Endres, *et al*.^21^ describe a drastic downregulation of mature ADAM17 at the cell surface upon stimulation with PMA, Soond, *et al*.^25^ observed an increase. We found both to be true for HEK293 and HeLa cells by looking at different time points after stimulation. PMA induces a rapid relocalisation of mature ADAM17 to the cell surface and a subsequent internalisation and degradation of the protease. Since HEK293 and HeLa cells showed slightly different rates of ADAM17 relocalisation, the underlying kinetics seems to depend on the cell type, but the overall tendency appears to be the same.

However, compared to unstimulated cells there were no differences in the localisation of ADAM17 upon stimulation with GPCR ligands, although shedding was induced. Since PMA artificially activates all diacylglycerol-dependent PKCs, many different intracellular signalling pathways are parallel activated which might not happen under physiological conditions. Taken together our data clearly demonstrate that PMA-induced relocalisation of ADAM17 does not represent a process directly linked to activation of shedding or might not be a physiological process at all. This PMA-induced dysregulation of ADAM17 localisation has to be taken into consideration when assessing previous studies or planning future experiments.

Nonetheless, the fact that PKC activity interferes with the regulation of ADAM17 localisation raises new questions for future studies. We conclude from our findings, that most mature ADAM17 is stored intracellularly and only a small amount is present at the cell surface. The regulatory processes and molecular machinery, which prevent mature ADAM17 from reaching the surface and which can be blocked by PKC activity, have yet to be found. This might be the mechanism that enables the cells to regulate the surface abundance of ADAM17 and thereby for example compensate the loss of surface ADAM17 due to internalisation or modulate the intensity of the shedding activity.

A regulator, which determines the fate of ADAM17 after internalisation in resting cells, was recently described. Phosphofurin Acidic Cluster Sorting Protein 2 (PACS-2) diverts ADAM17 away from degradation and instead promotes recycling of the protease^29^. Further studies are necessary to examine whether PMA-induced PKC activity also interrupts the function of PACS-2 or another regulator of ADAM17 internalisation and thereby promoting the degradation of the protease. Furthermore, the loss of mature ADAM17 after stimulation with PMA raises the question of why no new mature ADAM17 is generated, although there is plenty of immature ADAM17 in the ER. One explanation is that PMA-induced PKC activity might also block maturation. However, it was previously described that ADAM17 maturation is a slow process^25^. We showed that 24 hours after treatment with PMA the pool of mature ADAM17 had still not recovered. Additionally, an attempt to accelerate the recovery by overexpressing iRhom1, a limiting factor for ADAM17 forward trafficking, did not succeed. Nonetheless, a very slow maturation process, which has yet to be fully understood, would explain the need of an intracellular storage of mature ADAM17 (Fig. 6).

Since ADAM17 generates two potent initiators of immune response, the soluble IL-6 receptor and TNF-α, it represents a key component in the pathophysiology of autoimmune and chronic diseases^9^,^13^,^43^. Additionally, elevated EGFR signalling caused by excess ADAM17 activity is associated with cancer progression^1^. Hence, ADAM17 represents an important potential therapeutic target. However, efforts to create a specific small molecular inhibitor against ADAM17, which does not interfere with other metalloproteases, have so far failed^44^. Nevertheless, the fact that most of the mature ADAM17 is stored intracellularly, and that transport to the surface, where the shedding process takes place, seems to be tightly regulated, might provide a new opportunity to therapeutically target ADAM17 activity.

In summary, we report that activation of ADAM17-mediated shedding does not necessarily cause a change in localisation or degradation of the protease. However, PMA-induced PKC activity dysregulates the localisation of mature ADAM17 leading to a fast and drastic increase of its surface abundance, which is followed by internalisation and subsequent degradation. Overall, the localisation of ADAM17 seems to be tightly regulated and consists of four major steps (Fig. 6). The first step is the ER to Golgi trafficking of ADAM17 by iRhoms. This is followed by the maturation of the immature form in the trans-Golgi network. In a third step, a small amount of the intracellularly stored, mature protease is transported to the cell surface. The fourth and final step represents the clathrin-dependent internalisation and the subsequent recycling or degradation.

## Experimental Procedures

### Cells and Reagents

HEK293T and HeLa cells (DSMZ GmbH, Germany) were cultured in a humidified incubator at 37 °C with 5% CO_2_ in DMEM high-glucose (Sigma-Aldrich, Germany) containing 10% foetal calf serum (FCS), 100 mg/l streptomycin and 60 mg/l penicillin (DMEM 10%).

To detect ADAM17 the mouse anti-ADAM17 monoclonal antibodies A300D (Western blot) and A300E (flow cytometry) were used^45^,^46^,^47^. The following antibodies were obtained from commercial sources: anti-Flag M2 (F1804, Sigma-Aldrich, Germany), anti-β-actin C4 (sc-47778, Santa Cruz Biotechnology, USA), anti-ADAM10 SHM14 (352702, Biolegend, USA), anti-Transferrin Receptor ab84036 (Abcam, UK), allophycocyanin (APC)-conjugated goat anti-mouse IgG from Jackson Immuno Research Laboratories (Dianova GmbH, Germany), peroxidase conjugated secondary antibodies (Thermo Fisher Scientific, Germany), Alexa Fluor 488 conjugated donkey anti-mouse IgG (Thermo Fisher Scientific, UK), anti-mouse IgG DyLight 800 (Insight Biotechnology Limited, UK). Ikarugamycin (IK), genistein (GE), histamine, phorbol-12-myristate-13-acetate (PMA), Bisindolylmaleimide II (BimII), marimastat (Ma), and polyethylenimine (PEI) were obtained from Sigma-Aldrich (Germany). The inhibitors GW280264X (GW, selective for ADAM10 and ADAM17) and GI254023X (GI, selective for ADAM10) were synthesized by Iris Biotech (Marktredwitz, Germany). Ammonium chloride (NH_4_Cl) was obtained from Roth (Germany). The thrombin receptor agonistic peptide (TRAP-6 amid, H-2936) was obtained from Bachem (Germany).

### Western blotting

Samples were separated by SDS-PAGE under reducing conditions and transferred onto polyvinylidene difluoride (PVDF) membranes (Immobilon-P or Immobilon-FL for fluorophore-coupled antibodies, Millipore). Membranes were blocked with 5% BSA in TBS (50 mM Tris, 150 mM NaCl, pH 7.4), and subsequently incubated with primary antibodies in 0.1% Tween-TBS with 1% BSA and washed in 0.1% Tween-TBS. Bound primary antibodies were detected with peroxidase-conjugated goat anti-mouse or goat anti-rabbit antibodies using the ECL detection system SuperSignal West Pico (Thermo Fisher Scientific, Germany). For quantification of ADAM17 the fluorophore-coupled secondary antibody anti-mouse IgG DyLight 800 was used and the band intensity was measured with Image Studio Lite (LI-COR, USA).

### Shedding assays

For shedding assay 4 × 10^5^ HEK293 cells per well were seeded in 6-well plates. After 24 hours the cells were transfected with IL-1R_II_ fused to an alkaline phosphatase (AP) tag. For stimulation experiments with histamine, the histamine H_1_ receptor (H1R) was co-transfected. 24 hours after transfection, cells were left untreated, stimulated or stimulated and treated with inhibitors. 100 nM PMA, 30 μM TRAP-6 or 10 μM histamine were used for stimulation. Unstimulated cells were treated with solvent (DMSO). For inhibition, the following concentrations of compounds were used: against ADAM10 activity 1 μM of GI, against ADAM10 and ADAM17 activity 1 μM of GW, against PKC activity 5 μM BimII, against metalloprotease activity 10 μM marimastat (Ma). Cells were incubated for 30 minutes at 37 °C in DMEM containing the indicated stimulator and inhibitor. The shedding activity was assessed as described before^48^.

### Degradation experiments

For degradation experiments 4 × 10^6^ cells were seeded in 10 cm culture dishes. If necessary, cells were transfected 24 hours after seeding. The experiments were performed 48 hours after seeding. Cells were stimulated as described above. For stimulation with PMA cells were either incubated the whole indicated time with PMA or only for 5 minutes with an additional PBS washing step and a subsequent incubation in DMEM containing 5% FCS, 100 mg/l streptomycin and 60 mg/l penicillin (DMEM 5%). For stimulation with Histamine, cells were transfected with H1R. To inhibit lysosomal degradation or PKC activity, cells were treated with 50 mM NH_4_Cl or 5 μM BimII, respectively, during the experiments starting 30 minutes prior stimulation. After incubation at 37 °C in DMEM 5% for the indicated times cells were harvested and lysed in 1 ml lysis buffer (20 mM Tris, 150 mM NaCl, 2 mM EDTA, 0.5% Triton-X-100, 0.1% SDS, pH 7.4 and complete protease inhibitor mixture (Roche Applied Science, Germany)) for 30 minutes at 4 °C. To enrich ADAM17 30 μl Concanavalin A beads (Sigma-Aldrich, Germany) were added to 1 ml lysate with a protein concentration of 1mg/ml and incubated for 30 minutes at 4 °C. Afterwards the beads were washed five times with lysis buffer and prepared for Western blotting by heating them for 5 minutes at 95 °C in 60 μl 2.5x Lämmli buffer (6% SDS, 30% Glycerol, 5% β-mercaptoethanol, 150 mM Tris-HCl (pH 6.8) and 0.2% bromophenol blue).

### Flow Cytometry

For detection of cell-surface proteins by flow cytometry, HEK293 cells were stimulated as described above, washed in PBS and stained with primary antibodies diluted in FACS buffer (PBS, 0.5% BSA) for 1 hour. Cells were washed in FACS buffer and then incubated with fluorophore-conjugated mAb for 1 hour. After two washing steps in FACS buffer, cells were suspended in 300 μl FACS buffer and analysed by flow cytometry on a BD FACS Canto II or BD FACSCalibur. For analysis of DN-AP180, cells were transfected 48 hours prior flow cytometry analysis. Unstained cells were used as negative control. For staining of intracellular proteins, cells were permeabilised with 0.1% saponin in FACS buffer. All other steps remained the same.

### Surface biotinylation

For biotinylation of cell-surface proteins cells were washed three times with PBS (pH 7.4), once with PBS (pH 8, 4 °C) and were incubated with 5 ml of 0.25 mg/ml EZ-Link Sulfo-NHS-LC-Biotin (Perbio Science, Germany) in PBS (pH 8, 4 °C) for 30 minutes at 4 °C. Cells were harvested and lysed in lysis buffer. 2 mg biotinylated proteins were incubated with 40 μl streptavidin beads for 1 hour at 4 °C. Afterwards beads were washed three times with lysis buffer and three times with wash buffer (20 mM Tris (pH 7.4), 500 mM NaCl, 2 mM EDTA, 0.5% Triton-X-100). For Western-Blot analysis the beads were heated in 70 μl 2.5x Lämmli buffer.

### Pulse chase experiments

For pulse-chase experiments surface proteins were biotinylated as described before. After surface biotinylation cells were stimulated with 100 nM PMA or 30 μM TRAP-6, or were left unstimulated and were incubated in DMEM 5% at 37 °C. After indicated times cells were harvested and biotinylated proteins were enriched as described before. The cells, which were stimulated with PMA, were treated with PMA only for 5 minutes. Afterwards, these cells were washed with PBS and subsequently incubated in DMEM 5% without PMA. For inhibition of endocytosis pathways cells were treated with 200 μM genistein, 5 μM ikarugamyin or both starting 1 hour prior biotinylation.

### Peptide-substrate cleavage assay

For the activity assay the SensoLyte 520 TACEActivity assay kit (ANASpec Inc., USA) with the fluorogenic peptide OXL^TM^ 520/5-FAM (ADAM17 cleavage site from TNF-α) was used. 5 × 10^4^ HEK293 cells per well were seeded in a 96-well plate. 24 hours after seeding the cells were incubated in OPTI-MEM (Thermo Fisher Scientific, UK) with the peptide substrate for 30 minutes at 37 °C and the increase of fluorescence at 538 nm (Excitation: 485 nm) was monitored as baseline activity. Afterwards cells were treated with inhibitor and/or stimulator/solvent and the activity was monitored at 37 °C for one hour. The activity of the unstimulated/stimulated cells was normalised to their baseline activity respectively.

### Presentation of experimental data

Data are expressed as mean values ± standard deviation throughout the manuscript. Results of shedding assays are derived from at least three independent experiments. To determine statistical significance unpaired, two-tailed Student’s t-test was used (*p < 0.05, **p < 0.01, ***p < 0.001). For flow cytometry and western blots one out of at least three experiments with similar outcome is shown.

## Additional Information

**How to cite this article**: Lorenzen, I. *et al*. Control of ADAM17 activity by regulation of its cellular localisation. *Sci. Rep*. **6**, 35067; doi: 10.1038/srep35067 (2016).

## Supplementary Material

Supplementary Information
