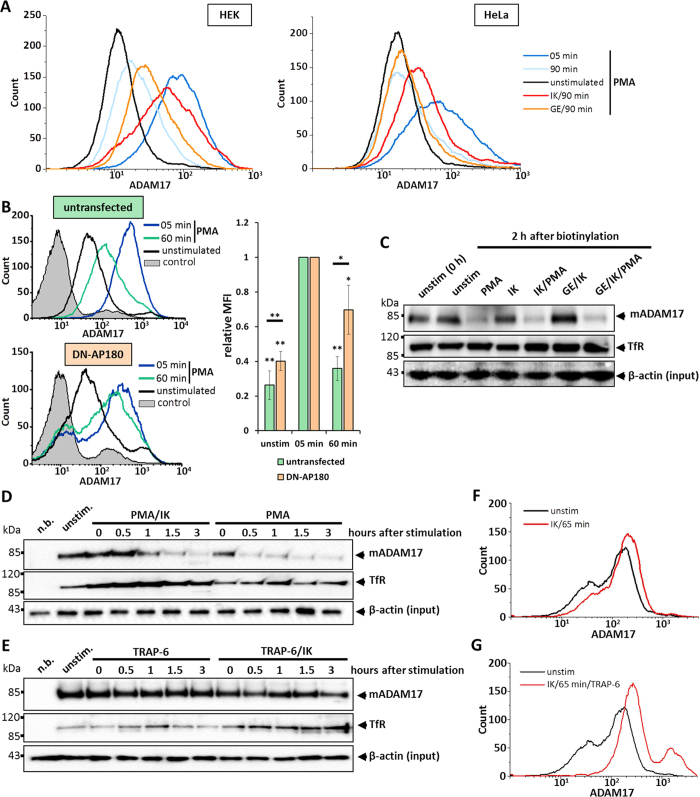# Erratum: Control of ADAM17 activity by regulation of its cellular localisation

**DOI:** 10.1038/srep37364

**Published:** 2016-11-21

**Authors:** Inken Lorenzen, Juliane Lokau, Yvonne Korpys, Mirja Oldefest, Charlotte M. Flynn, Ulrike Künzel, Christoph Garbers, Matthew Freeman, Joachim Grötzinger, Stefan Düsterhöft

Scientific Reports 6: Article number: 35067; 10.1038/srep35067 published online: 10 12 2016; updated: 11 21 2016.

This Article contains an error in panel B of Figure 4, where the 60 minute PMA stimulation results for ‘DN-AP180’ cannot be clearly seen. The correct Figure 4 appears below as Figure 1.

## Figures and Tables

**Figure 1:**