# Supplementary material for: Control of ADAM17 activity by regulation of its cellular localisation
Source: Sci Rep. 2016 Oct 12;6:35067. doi: 10.1038/srep35067 (PMC5059621; doi:10.1038/srep35067)
Supplement: Supplementary Information [file srep35067-s1.pdf]

Supplementary information

Control of ADAM17 activity by regulation of its cellular localisation

Inken Lorenzen[‡ 1], Juliane Lokau[‡ 1], Yvonne Korpys[‡], Mirja Oldefest[‡], Charlotte M Flynn[‡], Ulrike Künzel[§], Christoph Garbers[‡], Matthew Freeman[§], Joachim Grötzinger[‡], Stefan Düsterhöft[‡§] *

[‡]Institute of Biochemistry, Kiel University, Olshausenstr. 40, 24098 Kiel, Germany

[§]Sir William Dunn School of Pathology, South Parks Road, Oxford OX1 3RE, UK

[1]These authors contributed equally.

* To whom correspondence should be addressed: Dr. Stefan Düsterhöft, Dunn School of Pathology, South Parks Road, Oxford OX1 3RE, UK; Fax: +44 1865 275515; E-mail: stefan.duesterhoeft@path.ox.ac.uk

Suppl.Fig. 1

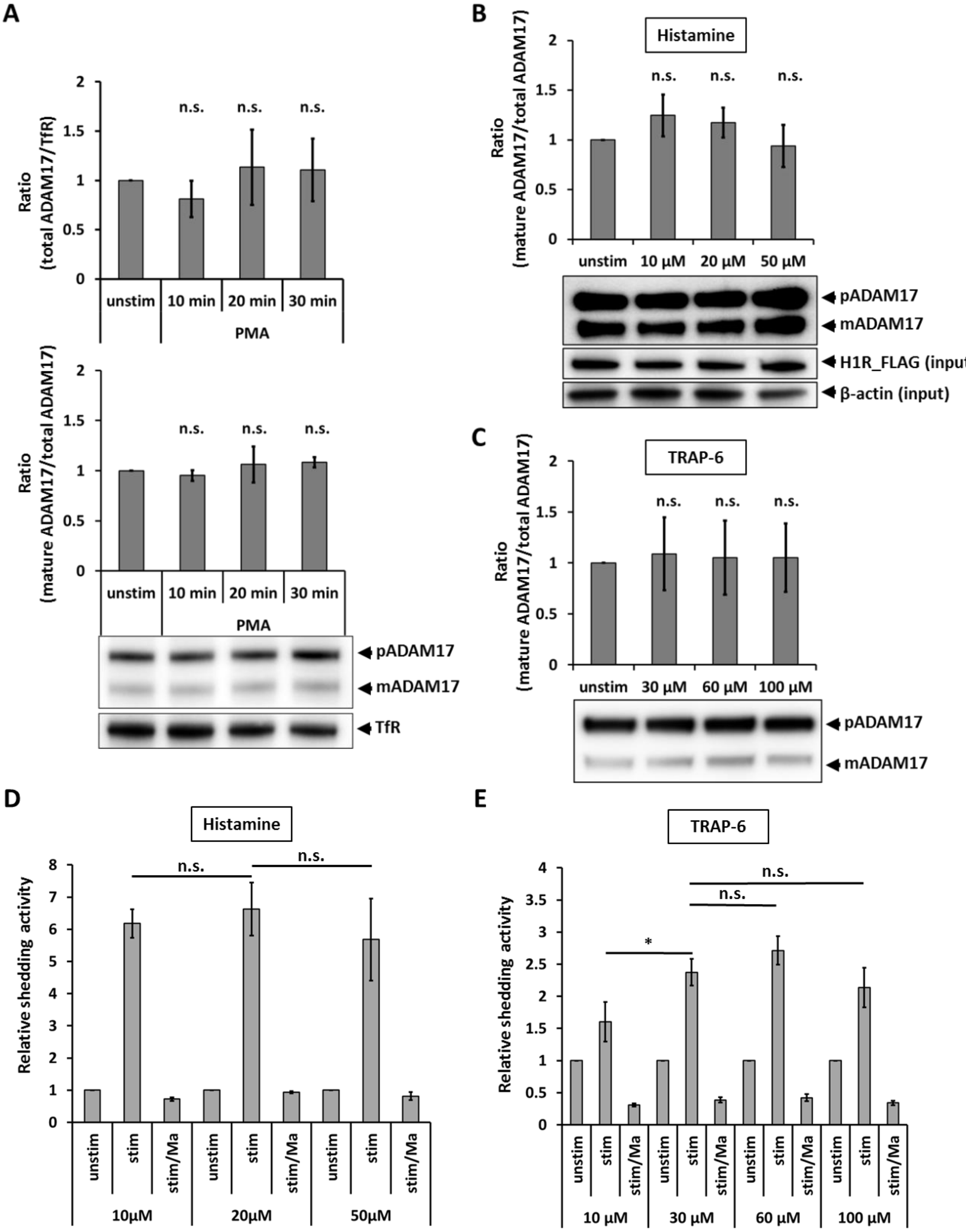

(A) - (C) Glycosylated proteins were enriched by precipitation with ConA-Sepharose and immunoblotted (n = 3). **(A)** Influence of PMA on mature ADAM17 (mADAM17) and its proform (pADAM17) were analysed in HEK293 cells 10, 20 or 30 minutes after PMA stimulation (100 nM). Amount of mature ADAM17, its proform and transferrin receptor (TfR) was measured by densitometry of immunoblots and indicated ratios were normalised to the unstimulated samples. The results are mean values of three independent experiments. **(B)** - **(C)** Cells were incubated with indicated concentrations of Histamine or TRAP-6 for 8 hours. Amount of mature ADAM17 and its proform was measured by densitometry of immunoblots and indicated ratios were normalised to the unstimulated samples. The results are mean values of three independent experiments. **(D)** - **(E)** HEK293 cells were transfected with AP_IL-1R$_{II}$ or transfected with AP_IL-1R$_{II}$ and H1R. Shedding activity was measured after a 30-minute treatment with solvent/DMSO (unstim), with a stimulator

(stim) or with a stimulator and an inhibitor. Indicated concentrations of Histamine or of TRAP-6 were used as stimulators. For inhibition cells were treated with 10 µM metalloprotease inhibitor marimastat (Ma). All values were normalised to the unstimulated values. n = 3; *p < 0.05.

Suppl.Fig. 2

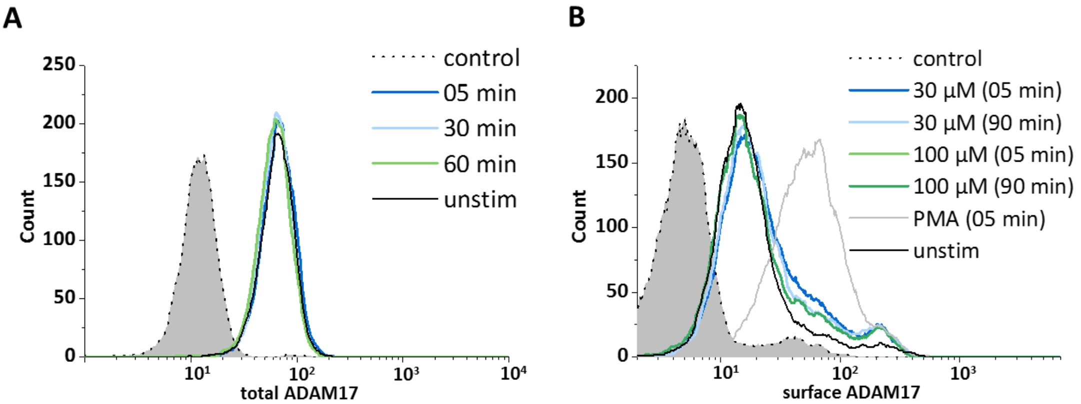

(A) HEK293 were either left unstimulated (unstim) or were stimulated with 100 nm PMA. At given time points cells were harvested, permeabilised and stained with anti-ADAM17 (A300E) for flow-cytometry analysis. Each depicted flow cytometry graph is one representative experiment of a set of n = 3. **(B)** HEK293 were either left unstimulated (unstim) or were stimulated with the indicated concentration of TRAP-6. At given time points cells were harvested and stained with anti-ADAM17 (A300E) for flow-cytometry analysis. Each depicted flow cytometry graph is one representative experiment of a set of n = 3.

Suppl.Fig. 3

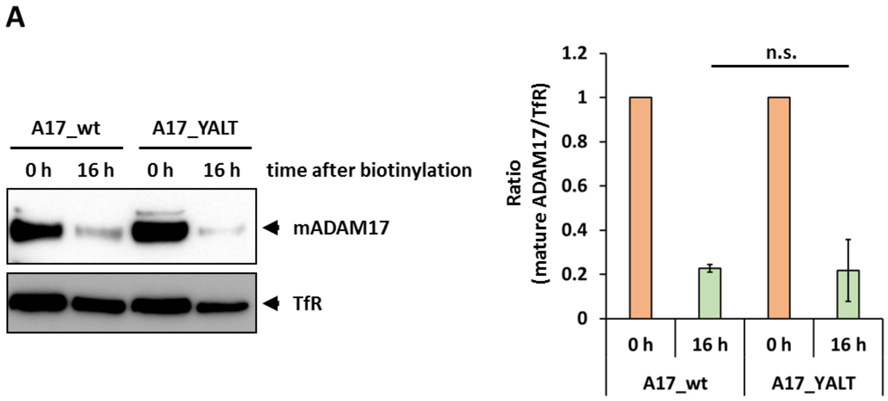

(A) HEK293 cells stably express wild-type ADAM17 (A17_wt) or the variant ADAM17_YALT (A17_YALT), both constructs contain a PC-tag. Cells were biotinylated and immediately harvested (0 h) or incubated for additional 16 hours (16 h) at 37 °C. To detect ADAM17 constructs on Western blot the anti-PC antibody HPC4 was used. The amounts of mature ADAM17 (mADAM17) and transferrin receptor (TfR) were measured by densitometry of immunoblots and ratio of both normalised to the 0 h sample respectively are shown (n = 3).
